# Quantifying barcodes of dendritic spines using entropy-based metrics

**DOI:** 10.1038/srep14622

**Published:** 2015-09-30

**Authors:** D. Viggiano, D. P. Srivastava, L. Speranza, C. Perrone-Capano, G. C. Bellenchi, U. di Porzio, N. J. Buckley

**Affiliations:** 1 Institute of Genetics and Biophysics “Adriano Buzzati Traverso”, CNR, Naples, 80131, Italy; 2 Department of Psychiatry, University of Oxford, Oxford, OX3 7JX, United Kingdom; 3 Department of Basic and Clinical Neuroscience, Institute of Psychiatry, Psychology and Neuroscience, King’s College London, London, SE5 8AF, United Kingdom; 4 Department of Physiology, Feinberg School of Medicine, Northwestern University, Chicago, Illinois, USA; 5 Department of Pharmacy, University of Naples “Federico II”, Naples, 80131, Italy; 6 Department of Medicine and Health Sciences, Univ. Molise, Campobasso, 86100, Italy

## Abstract

Spine motility analysis has become the mainstay for investigating synaptic plasticity but is limited in its versatility requiring complex, non automatized instrumentations. We describe an entropy-based method for determining the spatial distribution of dendritic spines that allows successful estimation of spine motility from still images. This method has the potential to extend the applicability of spine motility analysis to *ex vivo* preparations.

Known since the time of Cajal, dendritic spines are an interesting subdomain present in most neurons, thought to play a role in synaptic plasticity and memory storage^1^. In fact, interrogation of spine density (number of spines per unit length) has become one of the most widely used contemporary morphological correlates of plasticity in neurobiology^2^,^3^. However, this approach contrasts with two-photon microscopy data demonstrating, *in vivo*, that synaptic plasticity may occur without modification of the total number of spines. Empirical data have now documented that it is the number of ‘stable’ spines over time rather than the total number of spines that is reliable in studying synaptic plasticity^4^. Therefore, methods that measure spine motility/turnover promise better insight into the physiology of dendritic spines^5^. Unfortunately, these methods require complex instrumentations and a technically demanding setup, which limit the possibility to extend their application to high throughput systems. This has resulted in a widespread failure to address spatial distribution of spines. Here, we introduce the notion that spatial distribution of spines along a dendrite can be represented as a bar code, carrying information about the local network, which, in turn, is related to the spine motility. We present a new method to indirectly assess changes in spine dynamics, based on measures of entropy from information theory, which takes into consideration the spatial distribution of the spines rather than their abundance.

Entropy is a useful measure of the disorder and complexity in data series^6^, and similar quantities such as sample entropy, are better suited for short noisy time series^7^. It should be noted that the length of data series may, in fact, represent an important limitation when the aim is to calculate the amount of information (entropy) stored in the data; in the case of dendritic spines, the length of the data series is limited by the finite size of the dendrites and therefore their analysis require appropriate algorithms. Entropy (H) is a measure of the amount of information (classically measured in bits of information) required to describe a system. A sequence of random data shows high entropy, whereas a stream of very uniform, repetitive data shows low entropy (since less information is needed for their description).

## Methods

**Figure 1:**
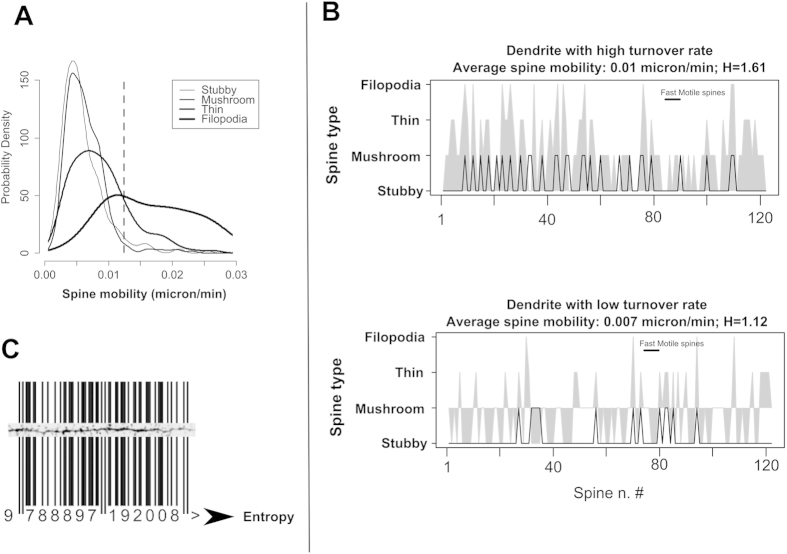
(**A**) Probability distribution function (or probability density) of the spine turnover rate for different spine types. The probability that a spine has a turnover in the interval between a and b is equal to the area under the probability density curve between a and b. (**B**) Graphical representation of the distribution of different spine types in dendrites with high turnover and low turnover of spines. The x-axis represents a sequence of spines in a segment of a representative dendrite (each spine is uniquely represented by its ordinal number in the dendrite); the vertical axis shows the type of each spine (stubby, thin, mushroom, filopodia). The black line represents whether the same spine has high or low motility. Dendrites with lower turnover rate (and lower number of motile spines; lower panel) show a different sequence of spine types along the dendrite (compare the grey outline in the upper and lower panel). (**C**) The distribution of spines along a dendrite, which is related to the spine motility, can be visualized as a bar code, carrying information. The amount of information stored can be read as Entropy (H) of distribution of the spines (in panel (**B**) the amount of entropy of the two dendrites is also reported and is lower in the case of dendrites with lower spine turnover). The number underlying the barcode is only for illustration purposes.

To explore if entropy can be a suitable approach to measure the distribution of dendritic spines, we used a transcranial two-photon imaging system to follow, in a time window of 30 min, identified spines of cortical neurons expressing GFP. Spine motility/turnover (we use these terms interchangeably within our text)was determined by measuring changes in spine length between consecutive time frames (see Supplementary Information (SI) for details). Spines were classified as fast or slow motile by applying a minimal motility threshold of 0.012 microns/min. This threshold was selected to distinguish between populations of thin spines and filopodia (as shown in Fig. 1A). The rationale is that filopodia are considered truly mobile protrusions, whereas stubby, mushroom and thin spines show lower levels of spine mobility.

To validate the method, we used a genetically modified animal with altered spine turnover, specifically Epac-2 knockout (ko) mice, and analyzed spine turnover of spine imaged using an *in vivo* two photon microscopy approach. Results concerning these animals with classical time-lapse counts have been published previously^8^. Breifly, nine four-week-old animals (three ko, three heterozygous and three wild type littermates, Epac2+/+, +/− or −/− respectivily) were used for the study. Epac2+/+, +/− or −/− mice were crossed with thy1-GFP-mice (Tg(Thy1-GFPM)2Jrs/J transgenic line (Jackson Labs)^9^ resulting in a subset of layer 5 cortical neurons expressing GFP. The dendritic spines on dendrites 15–40 μm from the pia surface were imaged *in vivo* with two-photon microscopy^8^. All animal procedures were approved by the Northwestern University Animal Care and Use committee (ACUC) and comply with the standards of the National Institutes of Health.

**Figure 2:**
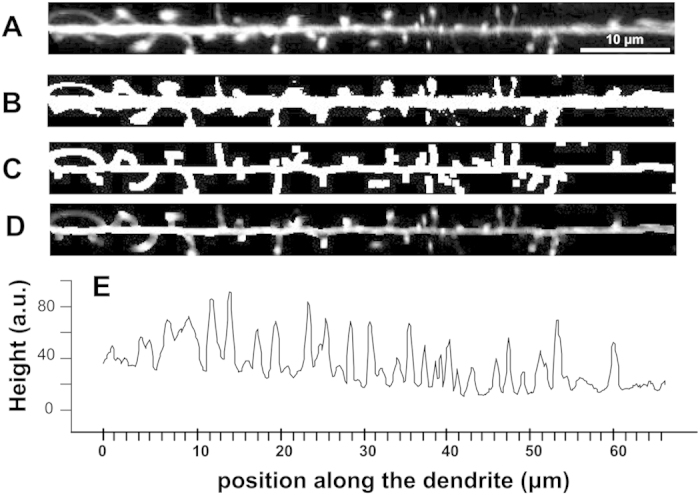
Algorithm to derive the profile plot of dendrites, which is then used to calculate the sample entropy of dendritic spines. (**A**) Using fluorescent images, individual dendrites are first selected and then straightened using the ‘straight’ tool of the ImageJ program. (**B**) The images are converted in B/W masks using the ‘Auto local threshold’ tool (parameters: method ‘mean’, radius 40). (**C**) The masks are then skeletonized in order to identify the main dendritic stem and the point of emergence of the spines. (**D**) This mask is then used to cut all the background on the original image, using an operation “AND” between the original image and the skeletonized one. (**E**) Finally, the resulting image is analyzed using the profile plot tool, thereby quantifying the position of each spine along the dendrite. The sample entropy of this profile (data sequence) is then calculated using a custom script in R environment (see Supplementary Methods).

Image stacks of dendritic spines were analyzed with ImageJ (http://rsbweb.nih.gov/ij/)using the following algorithm (Fig. 2): a single projection of the stacks was thresholded and converted into B/W images. The latter were skeletonized in order to obtain the main axis of the dendrite and of each spine. This skeleton was then used to select, on the original image, the dendrite with its spines, thereby deleting all the background noise. The resulting images of the dendrites and spines thus displayed homogeneous thickness. These images were then analysed using the ‘plot profile’ tool. This tool produces a plot representing, on the x-axis, the distance along the line along the main axis of the dendrite and on the y-axis, the average pixel intensity on a column perpendicular to that main axis. Since the images have been segmented, the main contribution to the value on the y-axis is given by the diameter of the dendrite (with its spines) and the profile plot can be read as the diameter of the dendrite (i.e. its height) as a function of the position along the dendrite (Fig. 2). The sequence of data was finally analysed in R environment (a free software environment for statistical computing and graphics) to estimate the sample entropy. Details about the calculation of samples entropy are reported in SI.

## Results

As shown in Fig. 1A, there is actually a certain overlap between spine populations (mushroom, stubby, thin, filopodia) with some filopodia behaving as low-motility spines and some thin spines showing higher motility. Therefore, to categorize spines in two groups, we used a threshold based on the point where the two distribution frequencies of thin spines and filopodia intersected as shown in Fig 1A. In P28 animals, we observed that the majority of the dendritic spines (77%) shows very low motility, in agreement with previous reports^5^ (Fig. 1A). Dendrites that, on average, show higher spine turnover had a higher number of motile spines (Fig. 1B). The turnover of spines along the dendrites causes a modification of the position of the spines and by comparing dendrites enriched in motile spines and dendrites with few motile spines, it was possible to identify a larger variety of spine types, apparently with a less regular spacing along the dendrite (Fig. 1B). This distribution of spines along a dendrite could be represented as a bar code (Fig. 1C) where the width and spacing of the lines represent width and spacing of the corresponding spines.

Conceptually, it is not possible to know the meaning of a code written in a bar-code (or a sequence of dendritic spines) without a decryption key. However, it is possible to know whether a collection of bar codes is actually storing some information, or only a random sequence of numbers, or if it is just a regular repetition of the same sequence of numbers. It is possible to read out this information (the amount of information stored in a collection of bar-codes) by calculating the entropy of the barcodes since entropy (according to information theory) is a measure of the amount of information needed to describe a specific sequence of data. Importantly, when we subject these data to entropy analysis we find that the amount of information (Sample Entropy, H) required to describe the distribution of the spines in the dendrites is lower in the case of dendrites with lower spine turnover (Fig. 1B). In other words, dendrites with higher spine turnover are expected to show less regular distribution of spines along the dendrite compared to dendrites with lower spine motility. Accordingly, in order to understand whether the relative proportion of unstable spines is decreased or increased, it is sufficient to measure the entropy of spine distribution in a single still image of the dendritic profile.

**Figure 3:**
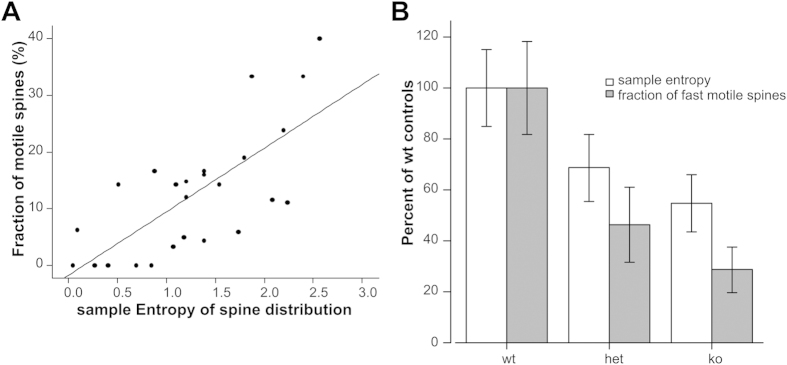
The Sample entropy of the position of dendritic spines is an estimate of the spine motility. (**A**) the sample entropy of spine position shows a linear relationship with the percentage of motile spines in a dendrite (Pearson correlation = 0.728, n = 25 dendrites, p ≪ 0.01). Sample entropy is not correlated with the density of dendritic spines (Pearson correlation −0.298, p = 0.092) nor with the length of the measured dendrites (Pearson correlation = 0.062, p = 0.730). (**B**) It is possible to characterize the difference in percentage of motile spines among different transgenic animals using sample entropy of spine distribution on still images (no requirement of time-lapse data). Epac 2 ko mice have a lower number of motile spines (F(2.33) = 10.7, p ≪ 0.01) and, correspondingly, lower sample entropy (F(2,23) = 3.73, p = 0.04); data represent mean ± SEM; n = 8 dendrites from 3 wt animals, 13 dendrites from 3 het animals and 15 dendrites from 3 ko animals. To compare sample entropy and number of motile spines data have been represented as per cent of wild type animals.

The results from Epac2 ko mice are shown in Fig. 3: the sample entropy of the spines is not related to the number of spines, but shows significant correlation with the number of motile spines. Importantly, pooling the data of dendritic spines from Epac2+/+, +/− or −/− mice, the sample entropy replicated the trend of the number of motile spines i.e. the decrease of spine motility seen in Epac2 −/− mice previously reproted^8^ was mirriored by a reduction of the entropy in Epac2 −/− animals.

## Discussion

Our analyses show that spine entropy can replace, at least in part, measurements of spine motility/turnover. In this study we have defined spine motility as extensive changes in length, including extensions, retractions (protrusive motility), and changes in spine morphology, such as enlargement and/or shrinkage (morphing)^10^. The morphology of spines often correlates with their dynamic properties, in that thinner spines and filopodia are highly dynamic displaying high levels of motility, while stubby or large mushroom shaped spines are more stable and thus less motile^11^,^12^,^13^. In general, protrusive motility has been associated with spine formation or elimination. For example, extensions from the dendrites search the surround neuropil for an appropriate partner. Once these protrusions find a pre-synaptic terminal, they stabilize, and their “protrusive motility” characteristic decrease^14^. Interestingly, the trafficking of synaptic proteins such as PSD-95 to synapses have also been shown to stabilize protrusive motility^15^. Conversely, spine morphing, or changes in spine morphology, has been associated with the maturation of synaptic contacts. This can be readily seen following activity-dependent stimulation, resulting in an enlargement of spine size^16^. Spine shrinkage can also be observed following activation of the Rap GEF, Epac2^10^ or following LTD^17^. Importantly, while spine shrinkage and spine elimination are related concepts, the former does not necessarily result in the later; spine shrinkage likely serves to destabilize existing synaptic connection without removing them.

Spine motility is highest during development, correlating to a periods when extensive synaptogenesis, and refinement of neural circuitry is occurring. However, spine motility can also be seen in adult animals, and has been suggested to be associated with the weakening of synaptic connections, switching of synaptic partner, strengthen of synaptic connections, and also formation or elimination of synapses^18^,^19^. Importantly, the level of motility seen in the adult brains is much less than that seen during development. Nevertheless, spine motility during adulthood represents a mechanism by which the refinement and tuning of neural circuits can occur.

Our approach, using simple image acquisition methods and automated analysis, is to readily allow comparison of multiple brain regions in high throughput systems. Such an approach could also be used to address the outstanding search for the ‘engram’ or memory trace, that is represented by the ensemble of synapses serving as physical representation of memory. Ideally, this would require the possibility to assess modifications of all brain synapses or dendritic spines in order to identify those that are involved in a memory trace. The method described here allows estimation of spine turnover *ex vivo*, thereby allowing the use of this metric in all brain regions of an animal, on fixed brain slices. The method would also work on fixed primary neurons *in vitro* imaged under conventional fluorescence microscopy.

Inferring indirect knowledge of dendritic spine motility/turnover from still images is currently of great interest because it is rapid and applicable to *ex vivo* preparations (brain slices) and, consequently can be applied to large number of brain regions in the same animal. In contrast, direct assessment of spine motility can only be studied using two photon microscopy and requires sophisticated *in vivo* preparations. In addition, *in vivo* imaging is very time consuming, does not allow observation of multiple brain locations at once, and is limited to cortical regions.

Our approach circumvents these limitations and offers a number of advantages: (i) no need for time series data (ii) possibility to study many different brain regions at the same time using *ex vivo* preparations (iii) automatic analysis of the data with attendant increase in speed and accuracy. The use of entropy as a surrogate of spine motility does have some drawbacks: firstly, it is not possible to identify which spines are motile, but entropy can be used to estimate the average motility of all spines along a dendrite. Second, as shown in Fig. 3A, the sample entropy of spine distribution is not only a function of the fraction of motile spines, but is dependent to a lesser degree on other unknown factors which generate some noise. Consequently, it is always advisable to take multiple measurements from multiple dendrites, in order to obtain a more realistic picture of the fraction of motile spines in the population of dendrites measured. Furthermore, in our experimental conditions, lasting only 30 minutes, the majority of the spines do not undergo drastic modifications and therefore the overall profile plot of the dendrite is not much modified. This leads to only minimal changes in dendritic entropy. To be able to observe time-course modifications of dendritic entropy (and hence of the average spine motility) longer time-course experiments are needed.

Our technique allows estimation of spine motility in fixed samples thereby enabling to address biological problems that are not solvable using *in vivo* preparations: specifically, it will be possible to characterize the probability distribution along dendrites of motile and non-motile spines, and distinguish differences among different neuronal types and different neuronal regions.

In conclusion, our data show that it is possible to estimate, using sample entropy of spine distribution, the average turnover rate of the dendritic spines. The main advantage of this approach is that there is no need of time-lapse images and it can be applied on fixed samples. The main disadvantage is the impossibility to derive the motility of a single spine. However, this limitation is counterbalanced by the possibility to design high throughput systems to study spine motility concurrently in large number of brain regions. The possibility to identify modifications of spine motility on a large number of brain regions will facilitate experiments designed to identify those synaptic sets that are directly involved in establishment of specific memories and, in the longer term, design of interventions to remove specific “bad” memories by deleting these synaptic sets.

## Additional Information

**How to cite this article**: Viggiano, D. *et al.* Quantifying barcodes of dendritic spines using entropy-based metrics. *Sci. Rep.* **5**, 14622; doi: 10.1038/srep14622 (2015).

## Supplementary Material

Supplementary Information
