# Supplementary material for: Quantifying barcodes of dendritic spines using entropy-based metrics
Source: Sci Rep. 2015 Sep 30;5:14622. doi: 10.1038/srep14622 (PMC4588562; doi:10.1038/srep14622)
Supplement: Supplementary Information [file srep14622-s1.doc]

**Quantifying barcodes of dendritic spines using entropy-based metrics**

**Viggiano D^1,6^ , Srivastava DP^3,4^, Speranza L^1^, Perrone-Capano C^5,1^, Bellenchi GC^1^, di Porzio U^1,*^, Buckley NJ^2,*^**

^1^Institute of Genetics and Biophysics "Adriano Buzzati Traverso", CNR, Naples, 80131, Italy

^2^Department of Psychiatry, University of Oxford, Oxford, OX3 7JX, United Kingdom

^3^Department of Basic and Clinical Neuroscience, Institute of Psychiatry, Psychology and Neuroscience, King's College London, London, SE5 8AF, United Kingdom

^4^Department of Physiology, Feinberg School of Medicine, Northwestern University, Chicago, Illinois, USA

^5^Department of Pharmacy, University of Naples “Federico II”, Naples, 80131, Italy

^6^Department of Medicine and Health Sciences, Univ. Molise, Campobasso, 86100, Italy

***Corresponding authors:**

Noel Buckley

Department of Psychiatry

University of Oxford

Warneford Hospital

OX3 7JX

Oxford, United Kingdom

email: noel.buckley@psych.ox.ac.uk

Umberto di Porzio

Institute of Genetics and Biophysics “Institute of Genetics and Biophysics "Adriano Buzzati Traverso", CNR

Via Pietro Castellino 111

80131

Naples, Italy

email: diporzio@igb.cnr.it

**Supplementary methods**

Surgical preparation for in vivo imaging

Mice (C57/Bl6) expressing GFP under the control of the thy1 promoter in a subset of cortical layer 5 neurons were used (Tg(Thy1-GFPM)2Jrs/J transgenic line (Jackson Labs); Feng. et al. Neuron, 28, 2000). Animals were 4 weeks old (P28) at time of imaging. Mice were anesthetized using 1.5% isoflurane, and a body temperature of 37°C maintained throughout the experiment. The skull was exposed, scrubbed and cleaned with ethanol and PBS. M1 motor cortex was identified according to stereotaxic coordinates. The skull over the motor cortex was thinned by using a high-speed dental drill (Osada) and then removed with forceps to create a small (1 x 1 mm) craniotomy. The skull was periodically bathed in saline to ensure the underlying cortex did not experience damage due to excessive heat during drilling. Neurons could be imaged up to 5 hours post-surgery with no apparent structural abnormalities. The craniotomy was filled with 3% agar and sealed with a No. 0 coverslip to ensure stability during imaging. A skull plate was then fastened to the skull using dental glue, and was then attached to metal base that connected directly to the microscope stage for imaging. During imaging, anaesthesia was maintained by a continuous supply of 1.5% isoflurane.

**Two-photon imaging**

A custom built two-photon laser scanning microscope was used for *in vivo* imaging (Jack Waters, Northwestern University). The microscope is based on an optical rail, and consisted of a coherent laser (Ti;sapphire laser) at a wavelength of 950 nm. Fluorescence z-stacks images were collected by photomultiplier tubes positioned in the epi-fluorescence beam path. The craniotomy over M1 was initially detected under low magnification (4X air lens; Zeiss), and areas with superficial dendrites were identified using a 40X water immersion lens (N.A. 0.8; Zeiss) under epi-fluorescence. The 40X lens with a digital zoom of X4, was used to image dendritic spines by two-photon imaging. Only dendritic spines on dendrites 15-40 µm from the pia surface were imaged, to minimize optical aberrations in the z-plane. Image acquisition was accomplished using a custom written script (Jack Waters, Northwestern University) in Labview (National Instruments). Z stacks were taken at 1 µm intervals every 5 minutes for 30 minutes. Up to 5 neurons per animal were imaged.

**Analysis of dendritic spine motility**

At each time point, images were acquired as Z stacks, taken at 1 µm intervals. Images were exported to ImageJ (http://rsbweb.nih.gov/ij/), and each time point aligned using the StackReg plugin, before being projected as a 2D maximum projection. Each time point were then arranged as a stack of 2D images (each image equal a single time point), and aligned using the StackReg plugin. Spines were then analyzed at each time point; movements of spines in the z dimension were not analyzed.

Spine motility was defined as the average change in length per unit time (micron/min) (Majewska and Sur PNAS 2003, 100:16024-16029). Images were exported to ImageJ (http://rsbweb.nih.gov/ij/), aligned using the StackReg plugin, and projected as a 2D maximum projection. Each time point was arranged as a stack of 2D images (each image constitutes a single time point), and spines were then analyzed at each time point; movements of spines in the z dimension were not analyzed. Dendritic spine length (base to tip) was measured for each spine at each time point in ImageJ, and the absolute difference between each time point was calculated, and averaged for each dendrite analyzed giving a motility index (micron/min). Only spines that were clearly visible were analyzed. In our experiments, no obvious rotational drift was seen (Srivastava et al. J Neurosci 2012, 32:11864-11878). Importantly, as measurements of spine motility were carried out at each time point and represented as the absolute difference between each time, we were not reliant on the alignment of the image stacks. However, if the StackReg plugin was unsuccessful in registering the, alignment was carried out manually using MetaMorph (Molecular Devices) (for more details see Srivastava et al. J Neurosci 2012, 32:11864-11878).

**Sample Entropy**

The sample entropy was calculated with the function “sample_entropy”, which is part of the R package 'pracma'. Sample entropy was introduced by Richman and Moorman (Am J Physiol Heart Circ Physiol 2000, 278:H2039-H2048) to quantify the regularity in a data series, without couning the self-matches. Many other algorithms to estimate entropy of a time series have been implemented, but in most cases the results depend on the length of the data, whereas sample entropy does not heavily depend on the time series length (other methods used to calculate entropy are the maximum likelihood estimator, the Mille-Madow and the NSB algorithm, the Bayesian estimators and the Chao Shen estimator, reviewed in Hausser and Strimmer, J Machine Learn Res 2009 10:1469-1484). In the case of the sample-entropy, the result is robust even in the presence of data corrupted by noise (Ramdani et al. Chaos 2009, 19:031231-031236).

The sample entropy is the negative logarithm of the probability that a subseries of length m that matches pointwise within a tolerance r also matches at the next point (Mei Z et al . Biomed Eng Online. 2013 12:101). The tolerance r and the matching length or embedding dimension m can be determined by an optimal method; however, most of the studies select m=2 and r= 0.2 x s.d. of the data, which is also used in the present work. However, further investigations are needed to explore possible improvements for the optimal selection of m and r in the case of calculations on dendrites. The algorythm follows these steps:

(1) Given a sequence of data of length n, {x1, x2, …, xn}, a subseries (template vector) of length m is defined:

Xi=[xi,xi+1,…,xi+m-1].

(2) the Chebyshev distance is calculated between all pairs of subseries Xi of length m:

d[Xi,Xj]

(3) The number of vector pairs of length m, with a distance smaller than the tolerance r is thus counted:

B = number of subseries of length m having d[Xi,Xj]<r

(4) steps 1-3 are repeated using template vectors of length m+1, thereby counting the number of vector pairs of length m+1 with a distance smaller than r

A = number of subseries of length m+1 having d[Xi,Xj]<r

**ImageJ macro**

/*

the background has been corrected with rolling ball (radius 6) on each slice to compensate for the difference in light intensity along the dendrite

*/

run("Subtract Background...", "rolling=6 stack");

/*

After background removal, a segmented line with a radius of 40 pxls was used to manually fit the dendritic shaft. Possible interfering fibers were manually deleted.

*/

run("8-bit");

run("Straighten..."); // the dendrite is straightened along the segmented line

/*

The image was (8-bit) duplicated. One copy was thresholded using a mean local threshold (radius 40 pixels) and skeletonized; the resulting skeleton was dilated two times

*/

run("Duplicate...")

run("Auto Local Threshold", "method=Mean radius=40 parameter_1=0 parameter_2=0 white");

run("Skeletonize");

run("Dilate");

run("Dilate");

/*

the resulting BW mask was used to retreive from the original image only the pixels corresponding to the skeletonized dendrite. This was obtained using an AND operator on the two images (the original one and the skeletonized one)

*/

imageCalculator("And create", img1, img2)

//img1 and img2 is the name of the two images (the original and the skeletonized

/*

the resulting straightened dendrite is again selected with the line tool, and the command “profile” executed. The resulting plot represented on the y axis a value proportional to the diameter of the dendritic shaft+spines

the orientation of the profile plot was set in order to have the start of the dendrite on the left

*/

**R script**

# code to generate fig 1 in the paper by

# Viggiano et al. "Quantifying barcodes of dendritic spines using entropy-based metrics"

# first load spine data.

nomefile<- choose.files(default='*.csv',caption='Select .csv file containing the spine turnover data', multi=F)

# File format (file type: CSV using TAB as separator for columns):

# col 1-label of the dendrite, col 2-animal genotype,

# col3-turnover, col4 - spine_type

data <- read.table(nomefile, sep = "\t", head=T)

x <- data[,3] #spine turnover

s_type <- data[,4] # spine type: 0=stubby, 1=mushroom; 2=thin, 3=filopodia

genotype <- data[,2] #genotype (1= wt, 0.5=het, 0=ko)

dendrite_n <- data[,1] #uniquely identifyes each dendrite with a progressive number

# now produce density plot Fig 1A

x11(); plot(density(x[s_type==0],probability=T),col='black',lty=1,lwd=1)

lines(density(x[s_type==1]),col='black',lty=2,lwd=2)

lines(density(x[s_type==2]),col='black',lty=3,lwd=3)

lines(density(x[s_type==3]),col='black',lty=4,lwd=4)

leg.txt <- c("stubby","mushroom","thin","filopodia")

legend(0.02,150,leg.txt,lty=c(1,2,3,4),lwd=c(1,2,3,4))

# Fig 1B

# first categorize spines as fast/slow motility using

# a threshold of 0.012 microns/min (based on the results of Fig.1A)

motility <- rep(0, length(s_type))

motility[x>0.012] <- 1

par(mfrow=c(2,1))

serie <- motility[genotype==1][1:122]

plot(s_type[genotype==1][1:122],type='l',col='gray')

lines(serie ,type='l')

serie <- motility[genotype==0][1:122]

plot(s_type[genotype==0][1:122],type='l',col='gray')

lines(serie ,type='l')

#now analyze the entropy of the spatial distribution of spines along dendrites.

# load package pracma, which is used to calculate the sample entropy

library(pracma)

## load files containing the dendrite profile.

# this is the .txt file generated by ImageJ

# the algorithm needs all dendrite profile .txt files to be

# stored in a single folder. You can then just select all dendrite profiles.

filename_all<- choose.files(default='*.txt',caption='Select all .txt files containing the profile plot',

multi=T)

num_dendrites <- length(filename_all)

# PARAMETERS

tau=4

edim <- 4

# initialize the variable denrtie_entropy where results are stored

dendrite_entropy <- data.frame(entropy_SE=vector(), name=vector())

for (dendrite in seq(1,num_dendrites)){

filename <- filename_all[dendrite]

data <-read.table(filename)

profile <- data[,2]

profile <-(round(profile/max(profile)* 15))

dendr_entropy [dendrite,1] <- sample_entropy(profile, edim = edim, r = 0.2*sd(profile), tau = tau)

}

dendr_entropy[,2] <- filename_all

# write the results of measured entropy in a text file for further analysis in statistical package

write.table(dendr_entropy, file = "entropy_file.txt", append = F,col.names = F)
